# Elementary exact calculations of degree growth and entropy for discrete equations

**DOI:** 10.1098/rspa.2016.0831

**Published:** 2017-05-03

**Authors:** R. G. Halburd

**Affiliations:** Department of Mathematics, University College London, Gower Street, London WC1E 6BT, UK

**Keywords:** algebraic entropy, discrete Painlevé equations, singularity confinement

## Abstract

Second-order discrete equations are studied over the field of rational functions C(z), where *z* is a variable not appearing in the equation. The exact degree of each iterate as a function of *z* can be calculated easily using the standard calculations that arise in singularity confinement analysis, even when the singularities are not confined. This produces elementary yet rigorous entropy calculations.

## 1. Introduction

We will consider second-order discrete equations such as

1.1 $$y_{n+1}+y_{n-1}=\frac{a_{n}+b_{n}y_{n}}{1-y_{n}^{2}},$$

where (*a*_*n*_) and (*b*_*n*_) are as yet undetermined sequences in C. One of the first approaches to finding integrable cases of discrete equations such as (1.1) was the singularity confinement test of Grammaticos *et al.* [1], which has been used to identify many discrete Painlevé equations [2]. The main idea, based on an analogy with the famous Painlevé property for differential equations, is to study the behaviour of iterates after *y*_*n*_ takes a singular value (e.g. 1 or −1 in the case of equation (1.1)). Generically, infinitely many future iterates will be infinite, but for some special choices of (*a*_*j*_) and (*b*_*j*_), the singularity will be confined.

Although it is well known that singularity confinement is not a sufficient condition for a discrete equation to be integrable (in particular, some equations with the property are known to exhibit chaotic behaviour), this property, appropriately interpreted in different contexts, is known to be necessary in order to ensure that several measures of complexity of a solution *y*_*n*_ grow slowly compared with solutions of generic equations. In this paper, we will show how one can use little more than the standard calculations one performs when looking for singularity confinement in order to calculate such a measure of complexity rigorously yet simply.

We begin by illustrating a standard minimal analysis of equation (1.1) from the point of view of singularity confinement. In order to analyse the iterates beyond a singularity of equation (1.1), we consider that for a fixed integer *n*, *y*_*n*−1_ takes an arbitrary finite value, say *k*, and *y*_*n*_=*θ*+*ϵ*, where *θ* is either 1 or −1 and *ϵ* is a small parameter. We then calculate the next few terms in the Laurent series in *ϵ* for the subsequent iterates. This gives

1.2 $$\begin{array}{rl} y_{n-1} & \;=k,\\ y_{n} & \;=\theta +\epsilon ,\;\theta =\pm 1,\\ y_{n+1} & \;=-\frac{a_{n}+\theta b_{n}}{2\theta }\epsilon^{-1}+O(1),\\ y_{n+2} & \;=-\theta +\frac{2\theta b_{n+1}-\theta b_{n}-a_{n}}{a_{n}+\theta b_{n}}\epsilon +O(\epsilon^{2})\\ \text{and}\;\;y_{n+3} & \;=\frac{a_{n}+\theta b_{n}}{2\theta }\left\{\frac{\left(a_{n+2}-a_{n})-\theta (b_{n+2}-2b_{n+1}+b_{n}\right)}{\theta (2b_{n+1}-b_{n})-a_{n}}\right\}\epsilon^{-1}+O(1), \end{array}\}$$

where we have assumed that *a*_*n*_≠±*b*_*n*_ and *a*_*n*_≠±(2*b*_*n*+1_−*b*_*n*_). In the limit *ϵ*→0, we see that $y_{n+1}=\infty$ and *y*_*n*+2_=−*θ*. Generically, *y*_*n*+3_ is also infinite unless

1.3 $$a_{n+2}-a_{n}=\theta (b_{n+2}-2b_{n+1}+b_{n}).$$

In order to confine all such singularities in this way, we demand that equation (1.3) holds for all *n* and for both choices *θ*=1 and *θ*=−1. Hence (1.3) decouples into the pair of linear equations *a*_*n*+2_−*a*_*n*_=0 and *b*_*n*+2_−2*b*_*n*+1_+*b*_*n*_=0 and equation (1.1) becomes

1.4 $$y_{n+1}+y_{n-1}=\frac{\alpha +\beta {\left(-1\right)}^{n}+(\gamma n+\delta )y_{n}}{1-y_{n}^{2}},$$

where *α*, *β*, *γ* and *δ* are constants. Equation (1.4) with *γ*≠0 is known to have a continuum limit to the second Painlevé equation and is often referred to as dP_II_, usually in the special case *β*=0. Equation (1.4) with *β*=0 first appeared in the work of Periwal & Shevitz [3] on exactly solvable string theories. It is the compatibility condition for a related linear problem and it is known to be a reduction of an integrable lattice equation [4].

Despite the success of this method in identifying a large number of discrete integrable equations, it is well known that some non-integrable equations also possess the singularity confinement property. For example, Hietarinta & Viallet [5] considered the equation

1.5 $$y_{n+1}+y_{n-1}=y_{n}+\frac{a}{y_{n}^{2}},$$

where *a* is a non-zero constant, which has the singularity confinement property, yet it exhibits chaotic behaviour. They suggested that the complexity of solutions as measured by algebraic entropy should be considered.

By considering *y*_0_ and *y*_1_ as variables, each future iterate *y*_*n*_ of an equation such as equation (1.1) is a rational function of *y*_0_ and *y*_1_. The algebraic entropy is a measure of how fast the degree *d*_*n*_ of *y*_*n*_ as a rational function of *y*_0_ and *y*_1_ grows. Specifically, the algebraic entropy is given by

$$\lim_{n\to \infty }\frac{\log d_{n}}{n}.$$

Integrability is associated with zero algebraic entropy, which corresponds to polynomial, as opposed to exponential, growth in *d*_*n*_. Algebraic entropy is related to ideas of complexity growth discussed in Arnol'd [6], Veselov [7] and Bellon & Viallet [8].

A practical method for calculating the algebraic entropy is to obtain a finite list of degrees *d*_*n*_ and then determine a generating function, from which the algebraic entropy can be determined simply [5]. Bellon [9] showed that discrete equations giving rise to a foliation of phase space by invariant curves have zero algebraic entropy; however, this result cannot be used to deduce the algebraic entropy of the discrete Painlevé equations. Rigorous methods based on a detailed analysis of the regularization of the equation through a sequence of blow-ups have also been applied [10,11]. Methods based on estimating the degree of cancelling factors have also provided rigorous bounds on the degree growth [12]. Studies of the cancellation and factorization properties of iterates have also been used in [13] to calculate algebraic entropy.

In this paper, we will consider *y*_0_ and *y*_1_ to be rational functions of an auxiliary parameter *z* and we will calculate the degree of all subsequent iterates *y*_*n*_ as functions of *z*. Rational functions of a single complex variable are much easier to deal with than rational functions of more than one variable. In particular, we do not need to consider blow-ups or cancellations to keep track of degrees. We will show how, with essentially no modification, standard singularity confinement calculations such as the one above can be used directly to determine the degrees of iterates. To calculate the degree of *y*_*n*_, the only extra information required from the equation is an analysis of some other singular initial conditions, which is often trivial. This measure of complexity has also been used in [14,15] where lower bounds on the degrees of iterates were obtained to show that many equations had exponential growth of degrees. In this paper, we are able to calculate the degrees exactly.

Studies of the images of straight-line initial conditions in projective space (corresponding to degree one initial conditions in our setting) have been used by Bellon & Viallet [8] and Viallet [16] to calculate degrees of iterates and algebraic entropy. In this paper, we emphasize the elementary (almost naive) calculations that are required to calculate the entropy rigorously and remark that these calculations are essentially the same ones that researchers have been doing in studying confinement.

Another advantage of this approach is that it allows us to study one-parameter families of solutions with lower complexity than the general solution. In this way, it can be used to look for integrable sub-cases of otherwise non-integrable equations or special solutions of integrable equations. It should be stressed that, although we are mostly considering the kind of calculations that appear in singularity confinement analysis, we do not require that the singularities be confined. These calculations merely provide the book-keeping for relating the various frequencies of certain singular values among nearby iterates.

This is yet another instantiation of the observation that most rigorous methods to estimate the growth of some measure of the complexity of a discrete equation ultimately demand an analysis of the singularities of the equation in the spirit of singularity confinement. Motivated by earlier work of Okamoto on the space of initial conditions for the (differential) Painlevé equations, Sakai [17] obtained a large number of discrete equations of Painlevé type by considering dynamical systems on ${CP}^{2}$ blown-up at nine points (equivalently ${CP}^{1}\times {CP}^{1}$ blown-up at eight points). The spaces so obtained are the spaces of initial conditions for the equations. It is well known that singularity confinement has an interpretation in terms of the resolution of singularities of mappings via a sequence of blow-ups. In [10], Takenawa used the Picard group associated with this sequence of blow-ups to show rigorously that the discrete Painlevé equations arising from Sakai's construction have zero algebraic entropy (in fact the degree growth is quadratic).

The degree of the *n*th iterate of a discrete equation relating three points can be shown itself to satisfy a recurrence with integer coefficients and a degree bounded in terms of the number of points that need to be blown-up to regularize the equation. So in principle one can determine a finite number of degrees to find this recurrence. However, quite a lot of work is needed to determine the number of blow-ups needed for a given equation. Also, iterating an equation to determine the degree when that degree grows exponentially is very difficult to do without a computer.

Singularity analysis along the lines of standard singularity confinement calculations also plays a key role in both Diophantine integrability [18] and the Nevanlinna approach to discrete integrability [19] in concluding the precise forms of certain integrable equations. In particular, it is invaluable in determining the precise form of coefficients in non-autonomous equations. In both these settings, one can obtain quite strong estimates on the degrees of various rational functions of the dependent variables in integrable equations, as shown in [19,18]. However, in order to obtain the precise forms of equations, including the dependence on the independent variable, it has been shown in the examples considered in [20–23] that singularity confinement is a necessary condition for slow growth of the relevant measure of complexity.

The measures of degree growth provided by Nevanlinna theory (the growth of the Nevanlinna characteristic), Diophantine integrability (growth of the height of solutions in a number field) and the growth of degrees as studied in this paper are discussed in [14] where a unifying theme is the use of singularity analysis to obtain lower bounds for complexity growth precise enough to detect exponential growth. In particular, the singularity confinement calculations in each setting are illustrated in detail to emphasize their similarities and differences. However, the analysis in this paper appears to be by far the simplest application of confinement to obtain a rigorous and precise measure of complexity.

## 2. Exact calculations of degrees

There are two equivalent characterizations of the degree of a rational function of a single complex variable *z*. Let *R*(*z*)=*P*(*z*)/*Q*(*z*), where *P* and *Q* are polynomials with no common factors. Then the degree of *R* is given by deg(R)=max{deg(P(z),deg(Q(z)}. However, for our purposes it is most practical to view *R* as a map from the extended complex plane ${CP}^{1}=C\cup \{\infty \}$ to itself. Let *a* be any number in the extended complex plane. Then the deg(*R*) is the number of pre-images of *a* in ${CP}^{1}$ counting multiplicities. For example, the degree of the rational function

$$R(z)=\frac{2z^{5}-4z^{4}+2z^{3}+z+1}{z{\left(z-1\right)}^{2}}=\frac{z+1}{z{\left(z-1\right)}^{2}}+2z^{2}$$

is five. The five pre-images of ∞ under *R*, listed according to multiplicity, are 0,1,1,∞,∞.

### (a) dP_II_

In this section, we will use the calculation (1.2) to relate the number of pre-images of 1, −1 and ∞ of different iterates *y*_*n*_ for *dP*_II_, equation (1.4). Suppose that *y*_*n*_(*z*) has a *θ*-point of multiplicity *p* at *z*=*z*_0_, where *θ*=±1. Then *y*_*n*_(*z*)=*θ*+*ϵ*, where *ϵ*=(*z*−*z*_0_)^*p*^*f*(*z*), where *f* is analytic at *z*_0_ and *f*(*z*_0_)≠0. Furthermore, assume that *y*_*n*−1_ takes some finite value *k* at *z*=*z*_0_. We assume that *θ*(*α*+*β*(−1)^*n*^)+(*γn*+*δ*)≠0, which is always true for sufficiently large *n*. As *z* tends to *z*_0_ we have

2.1 $$\begin{array}{rl} y_{n-1} & \;=k+o(1),\\ y_{n} & \;=\theta +\epsilon ,\\ y_{n+1} & \;=-\frac{1}{2}[\theta (\alpha +\beta {\left(-1\right)}^{n})+(\gamma n+\delta )]\epsilon^{-1}+O(1),\\ y_{n+2} & \;=-\theta +\frac{\gamma n+2\gamma +\delta -\theta (\alpha +\beta {\left(-1\right)}^{n})}{\left[\theta (\alpha +\beta {\left(-1\right)}^{n})+(\gamma n+\delta )\right]}\epsilon +O(\epsilon^{2})\\ \text{and}\;\;y_{n+3} & \;=O(1). \end{array}\}$$

Note that this is exactly the same calculation as (1.2) with *a*_*n*_=*α*+*β*(−1)^*n*^ and *b*_*n*_=*γn*+*δ*, apart from the ‘*o*(1)’ term in the expression for *y*_*n*−1_, which plays no role in the calculation.

We will assume that *θ*(*α*+*β*(−1)^*n*^)+(*γn*+*δ*)≠0 and *γn*+2*γ*+*δ*−*θ*(*α*+*β*(−1)^*n*^)≠0 for all *n*≥1. Note that these conditions are automatically satisfied for sufficiently large *n*, so by a translation in *n*, this condition can be satisfied if we provide initial conditions at a large value of *n*, rather than at *n*=0.

We see that, at any point *z*_0_ where *y*_*n*−1_ and *y*_*n*_ are both finite, then *y*_*n*+1_(*z*_0_) can only be infinite if *y*_*n*_(*z*_0_)=±1. Furthermore, the calculation (2.1) shows that in such a situation, the iterates *y*_*n*_(*z*_0_),*y*_*n*+1_(*z*_0_),*y*_*n*+2_(*z*_0_) take the values ±1,∞,∓1 with the same multiplicity and the next iterate is finite at *z*_0_. The only extra information that we require is to understand what happens at points where one or more of the initial conditions has a pole. To simplify the situation, we will consider the initial conditions *y*_0_(*z*)=*Az*+*B* and *y*_1_(*z*)=*Cz*+*D*, where *A*,*B*,*C* and *D* are constants and *AC*≠0. So the only poles of *y*_0_ and *y*_1_ are the simple poles at z=∞. From equation (1.4), we see that as z→∞,

$$y_{2k}(z)={\left(-1\right)}^{k}Az+O(1)\;\mathrm{and}\;y_{2k+1}(z)={\left(-1\right)}^{k}Cz+O(1).$$

Hence each iterate has a simple pole at z=∞.

**Figure 1.**
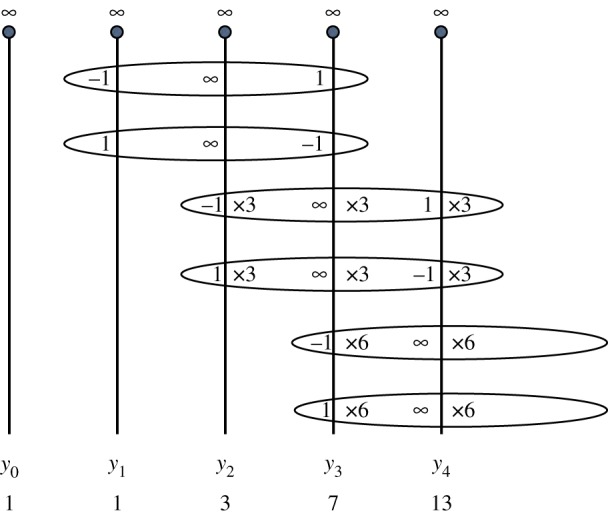
Calculating the degrees of the first few iterates of equation (1.4). (Online version in colour.)

In figure 1, each vertical line represents a copy of ${CP}^{1}$, which is the domain of the corresponding *y*_*n*_ indicated beneath it. The point at infinity is indicated at the top of the line and the ‘∞’ indicates that *y*_*n*_ has a simple pole there. As *y*_1_ has degree one, it has a single 1-point (of multiplicity one). This gives rise to a simple pole of *y*_2_ and a −1-point of *y*_3_. Similarly, there is a single −1-point of *y*_1_ giving rise to a simple pole of *y*_2_ and a 1-point of *y*_3_. Hence, there are exactly three (simple) poles of *y*_2_ (including the pole at infinity) and so the degree of *y*_2_ is three.

As *y*_2_ has degree three, it must have exactly three 1-points, counting multiplicities. In principle, this could be three simple 1-points or a 1-point of multiplicity three, etc. Now each such 1-point gives rise to the same number of infinities (i.e. poles) of *y*_3_, counting multiplicities. So the three 1-points of *y*_2_ generate three infinities of *y*_3_ and similarly the three −1-points of *y*_2_ generate three infinities of *y*_3_. Together with the simple pole at z=∞, we see that *y*_3_ has seven infinities and hence it has degree seven. Therefore, *y*_3_ has seven 1-points. One of these points comes from the −1 point of *y*_1_. So there are six ‘new’ 1-points. We introduce the notion *N*_*n*_ to describe new 1-points in this context. Apart from the simple pole at z=∞, *y*_4_ has *N*_3_=6 infinities generated by these 1-points and another *N*_3_=6 infinities generated by the new −1-points of *y*_3_. Hence the degree of *y*_4_ is 13.

Note that, for *n*>0, *y*_*n*_(*z*_0_) can only equal one as part of a sequence 1,∞,−1 or −1,∞,1. In the first case, we have called the 1-point ‘new’ as it is the beginning of the sequence. In the latter case, we call the 1-point ‘old’ as it is part of a sequence that began two steps earlier.

**Figure 2.**
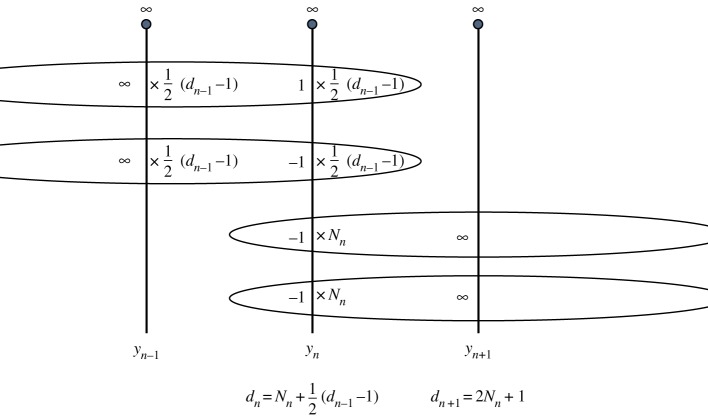
Calculating the degrees of the *n*th iterate of equation (1.4). (Online version in colour.)

The general case is illustrated in figure 2. We calculate the degree *d*_*n*+1_ of *y*_*n*+1_ by counting the pre-images of ∞. Now *y*_*n*+1_ has *N*_*n*_ infinities generated from the new 1-points of *y*_*n*_ and another *N*_*n*_ from the new −1-points of *y*_*n*_. Together with the simple pole at z=∞, we have

2.2 $$d_{n+1}=2N_{n}+1.$$

Also, the number of old 1-points of *y*_*n*_ is half the number of infinities of *y*_*n*−1_ in the finite plane (the other half generate the old −1-points of *y*_*n*_). Including the pole of *y*_*n*−1_ at infinity, we see that the number of old 1-points of *y*_*n*_ is (*d*_*n*−1_−1)/2. So the degree *d*_*n*_ of *y*_*n*_ expressed as the number of pre-images of 1 is

2.3 $$d_{n}=N_{n}+\frac{1}{2}(d_{n-1}-1).$$

Eliminating *N*_*n*_ from equations (2.2) and (2.3) gives *d*_*n*+1_−2*d*_*n*_+*d*_*n*−1_=2. Using the initial conditions *d*_0_=*d*_1_=1, we have

2.4 $$d_{n}=\frac{n(n-1)}{2}+1.$$

This obviously corresponds to zero algebraic entropy. For more general initial conditions, the poles of *y*_0_ and *y*_1_ can give rise to a string of poles of bounded multiplicity at the corresponding points of future iterates. However, we are still led to an equation in which *d*_*n*+1_−2*d*_*n*_+*d*_*n*−1_ is a bounded function of *n*, giving growth that it at most quadratic in *n*.

It is important to emphasize that this kind of reasoning in which we use pre-images of singular points to relate the degrees of different iterates does not rely explicitly on confinement, but it does use the kind of singularity analysis that one carries out in the context of studying singularity confinement. For example, if *a*_*n*_ and *b*_*n*_ are generic functions of *n*, then no singularity will be confined at any point. In this case, a pole of some iterate will arise at a point *z*_0_ if the two previous iterates are both finite at *z*=*z*_0_ if and only if the second value is *θ*=±1. This gives rise to an infinite sequence of iterates of the form θ,∞,−θ,∞,θ,∞,−θ,∞,θ,∞,…. If we start with the same initial conditions *y*_0_(*z*)=*Az*+*B* and *y*_1_(*z*)=*Cz*+*D*, then again every subsequent iterate will have a simple pole at z=∞ and every pole in the finite plane must arise in a sequence of the form just described. So for *n*>0, the only poles of *y*_*n*+1_ apart from the simple pole at infinity arise from each of the +1- and −1-points of *y*_*n*_. In terms of degrees, there are 2*d*_*n*_ such points, so the degrees satisfy *d*_*n*+1_−1=2(*d*_*n*_−1), i.e. *d*_*n*+1_=2*d*_*n*_−1, *n*≥1. Using *d*_1_=1, we have *d*_*n*_=2^*n*^−1. Hence the entropy is log⁡2.

For non-generic choices of the coefficients *a*_*n*_ and *b*_*n*_, it is known that there are infinitely many opportunities to confine the singularities of equation (1.1) by choosing appropriate (*a*_*n*_) and (*b*_*n*_). Only those equations that confine at the earliest opportunity appear to be integrable and have zero algebraic entropy [24]. In [25], this phenomenon is called *late* as opposed to the *infinitely late* confinement just discussed. Knowing where each type of singularity confines (or knowing that it does not confine at all) is enough to calculate the degrees for given initial conditions.

For special initial conditions, the degree growth of solutions of equation (1.4) can be slower than quadratic. In the simplest case, let us again take *y*_0_ and *y*_1_ to be degree one rational functions. Without loss of generality, we take *y*_0_(*z*)=*z*. In general, the simple pole of *y*_0_ at z=∞ and the simple 1-point and −1-point of *y*_1_ will force *y*_2_ to have exactly three simple poles and hence the degree of *y*_2_ would be three. We could prevent the pole at z=∞ of *y*_0_ from producing a pole at z=∞ of *y*_2_ by insisting that *y*_1_ is either −1 or 1 at z=∞. If $y_{1}(\infty )=-1$ and $y_{2}(\infty )$ is finite then

$$y_{1}(z)=-1+\frac{\alpha -\beta -\gamma -\delta }{2z}+O\left(\frac{1}{z^{2}}\right),$$

as z→∞. We can then force the degree of *y*_2_ to be one by choosing *y*_1_ to have a pole at *z*=1 and *y*_1_(−1) to be finite. In a sense, we are choosing the −1 point of *y*_0_ to be old and the 1-point to be new in the way described above. This uniquely specifies *y*_1_ to be

2.5 $$y_{1}(z)=\frac{f_{0}-z}{z-1},\;f_{0}=1+\frac{\alpha -\beta -\gamma -\delta }{2}.$$

It is straightforward to verify that if *γ*=2*α* then the solution *y*_*n*_ of equation (1.4) with the initial conditions *y*_0_(*z*)=*z* and *y*_1_(*z*) given by (2.5) also solves the discrete Riccati equation

2.6 $$y_{n+1}=\frac{f_{n}-y_{n}}{y_{n}-1},\;f_{n}=1-\frac{(2n+1)\alpha +\beta {\left(-1\right)}^{n}+\delta }{2}.$$

As *y*_*n*+1_ is a Möbius transformation of *y*_*n*_, we see that the degree of all iterates is one, so there is no degree growth at all. Other special initial conditions produce solutions that can be expressed in terms of solutions of discrete linear equations. In this way, by considering the growth of solutions of one-parameter solutions (the parameter being *z*), we can identify simpler solutions. (Integrable) discrete Riccati equations are linearizable. Another way in which we are lead to equation (2.6) is by demanding that a solution *y*_*n*_(*z*) of equation (1.4) only have singularities of the form 1, ∞, −1 and none of the form −1, ∞, 1.

In fact, if for some choice of *θ*=±1, we demand that a non-constant in *z* solution *y*_*n*_(*z*) of equation (1.1) only has singularities of the form *θ*, ∞, −*θ*, then we can find solutions governed by the discrete Riccati equation

$$y_{n+1}=\frac{f_{n}-\theta y_{n}}{y_{n}-\theta },$$

where *a*_*n*_ and *b*_*n*_ have the special form

$$a_{n}=\theta (f_{n-1}-f_{n})\;\mathrm{and}\;b_{n}=2-f_{n-1}-f_{n},$$

for some sequence *f*_*n*_. In this way, slow growth (or in this case, non-growth) of the degree of iterates singles out special integrable sub-classes of solutions of otherwise non-integrable equations.

### (b) An example of Hietarinta & Viallet

Now we turn to the example of Hietarinta & Viallet [5], equation (1.5). The only way that an iterate can become infinite starting from finite initial values is if the previous iterate has a zero. To this end, suppose that *y*_*n*_(*z*) has a zero of multiplicity *p* at *z*=*z*_0_. Then, *y*_*n*_=(*z*−*z*_0_)^*p*^*f*(*z*)=:*ϵ*, where *f* is analytic at *z*_0_ and *f*(*z*_0_)≠0. Suppose further that *y*_*n*−1_ has the finite value *k* at *z*_0_. Then as *z*→*z*_0_ we have

2.7 $$\begin{array}{rl} y_{n-1} & \;=k+\eta ,\;\eta =o(1),\\ y_{n} & \;=\epsilon ,\\ y_{n+1} & \;=a\epsilon^{-2}-k-\eta +\epsilon ,\\ y_{n+2} & \;=a\epsilon^{-2}-k-\eta +O(\epsilon^{4}),\\ y_{n+3} & \;=-\epsilon +O(\epsilon^{4})\\ \text{and}\;\;y_{n+4} & \;=k+o(1). \end{array}\}$$

To summarize, if *y*_*n*−1_(*z*_0_) and *y*_*n*_(*z*_0_) are finite but *y*_*n*+1_(*z*_0_) is not, then *y*_*n*_ has a zero of some multiplicity *p* at *z*_0_, *y*_*n*+1_ and *y*_*n*+2_ both have poles of multiplicity 2*p* at *z*_0_ and *y*_*n*+3_ again has a zero of multiplicity *p*. Also, *y*_*n*+4_ is finite at *z*_0_. The fact that there are many more poles compared with zeros is the source of the positive entropy (and ultimately the non-integrability) of this equation.

**Figure 3.**
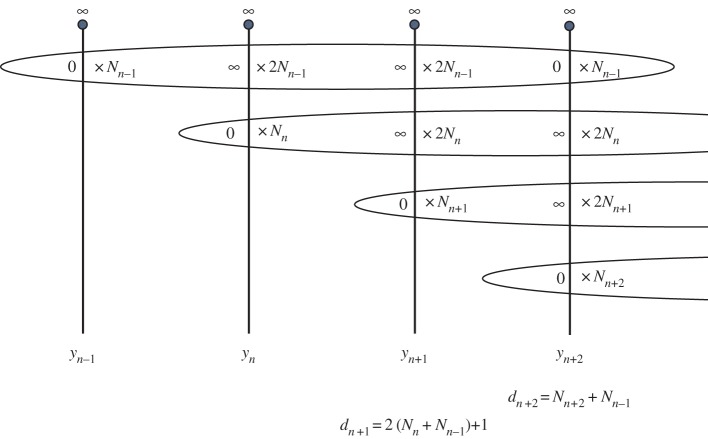
Calculating the degrees of the *n*th iterate of equation (1.5). (Online version in colour.)

We again choose initial conditions *y*_0_=*Az*+*B* and *y*_1_=*Cz*+*D*. If *AC*(*A*−*C*)≠0, then all iterates will have a simple pole at z=∞. We calculate the degree *d*_*n*_ of *y*_*n*_ with the aid of figure 3. Here, *N*_*n*_ denotes the number of ‘new’ zeros of *y*_*n*_, i.e. those zeros at the beginning of a sequence of the form $0,\infty^{2},\infty^{2},0$. The only poles of *y*_*n*+1_ in the finite complex plane come from sequences that began from new zeros of *y*_*n*_ and new zeros of *y*_*n*−1_. Recalling that the poles have twice the multiplicity of these zeros, and including the simple pole at z=∞, gives

$$d_{n+1}=2(N_{n}+N_{n-1})+1.$$

Next we calculate the degree of *y*_*n*+2_ as the number of pre-images of 0. Each of the old zeros of *y*_*n*+2_ comes from a new zero of *N*_*n*−2_. So

$$d_{n+2}=N_{n+2}+N_{n-1}.$$

Substituting *N*_*n*_+*N*_*n*−1_=(*d*_*n*+1_−1)/2 and *N*_*n*+2_+*N*_*n*−1_=*d*_*n*+2_ in

$$(N_{n}+N_{n-1})-(N_{n}+N_{n-3})+(N_{n-2}+N_{n-3})-(N_{n-1}+N_{n-2})=0$$

gives

$$d_{n+1}-3d_{n}+d_{n-1}=1.$$

Together with the initial conditions *d*_0_=*d*_1_=1, we find

$$d_{n}=\frac{\sqrt{5}-1}{\sqrt{5}}{\left(\frac{3+\sqrt{5}}{2}\right)}^{n}+\frac{\sqrt{5}+1}{\sqrt{5}}{\left(\frac{3-\sqrt{5}}{2}\right)}^{n}-1.$$

It follows that the entropy is

$$\ln\left(\frac{3+\sqrt{5}}{2}\right).$$

This value of the algebraic entropy of equation (1.5) was also obtained rigorously by Takenawa [10,11] after 14 blow-ups of ${CP}^{1}\times {CP}^{1}$.

### (c) dP_I_

Consider the equation

2.8 $$y_{n-1}+y_{n}+y_{n+1}=\frac{\alpha n+\beta }{y_{n}}+\gamma ,$$

where *α*, *β* and *γ* are constants. This equation first appeared in 1939 in the work of Shohat [26] on orthogonal polynomials. It later appeared in gauge field theory [27] and quantum gravity [28–30]. By considering the sequence beginning *y*_*n*−1_=*k*+*o*(1) and *y*_*n*_=*ϵ*, we find *y*_*n*+1_=(*αn*+*β*)/*ϵ*+*O*(1), *y*_*n*+2_=−(*αn*+*β*)/*ϵ*+*O*(1), *y*_*n*+3_=−*ϵ* and *y*_*n*+4_=*O*(1). The sequence of singular values here is similar to that of the previous example except that now the poles have the same multiplicity as the zeros. Starting from initial conditions *y*_0_=*Az*+*B* and *y*_1_=*Cz*+*D*, where *AC*(*A*+*C*)≠0, we see that each subsequent iterate has a simple pole at z=∞. The analysis is very similar to the previous example except we now have *d*_*n*+1_=*N*_*n*_+*N*_*n*−1_+1 and *d*_*n*+2_=*N*_*n*+2_+*N*_*n*−1_. Eliminating *N*_*n*_ we find the initial value problem *d*_*n*+1_−2*d*_*n*_+*d*_*n*−1_=1, *d*_0_=*d*_1_=1. So the degree of *y*_*n*_ is

$$d_{n}=\frac{n(n-1)}{2}+1.$$

### (d) dP_III_

We will study the integrable discrete equation dP_III_, which has the form

2.9 $$y_{n-1}y_{n+1}=b_{+}b_{-}\frac{\left(y_{n}-a_{+}q^{2n})(y_{n}-a_{-}q^{2n}\right)}{\left(y_{n}-b_{+})(y_{n}-b_{-}\right)},$$

where *a*_+_≠*a*_−_ and *b*_+_≠*b*_−_ are constants. Equation (2.9) was first identified in the seminal paper [2] by Ramani *et al*. Equation (2.9) has several routes into singularity. One kind of singularity arises when *y*_*n*−1_ is finite and *y*_*n*_ is either *b*_+_ or *b*_−_. Another kind of singular behaviour arises when *y*_*n*_ is either *a*_+_*q*^2*n*^ or *a*_−_*q*^2*n*^. This forces either *y*_*n*−1_ or *y*_*n*+1_ to be zero. Another route into singularity from finite values is when *y*_*n*−1_ vanishes.

For all sufficiently large *n*, *a*_±_*q*^2*n*^ is neither *b*_+_ nor *b*_−_. If *y*_*n*−1_(*z*_0_)=:*k* is non-zero and finite and *y*_*n*_ has a *b*_±_-point of multiplicity *p* at *z*_0_, then for generic *k*, *y*_*n*+1_ has a pole of multiplicity *p* at *z*_0_ and *y*_*n*+2_ has a *b*_∓_-point of multiplicity *p*. The next iterate is finite and non-zero. Similarly, if *y*_*n*−1_(*z*_0_)=:*k* is non-zero and finite and *y*_*n*_ has a *a*_±_*q*^2*n*^-point of multiplicity *p* at *z*_0_, then for generic *k*, *y*_*n*+1_ has a zero of multiplicity *p* at *z*_0_ and *y*_*n*+2_ has a *b*_∓_*q*^2(*n*+1)^-point of multiplicity *p*. The next iterate is finite and non-zero. In this way, both of these singular behaviours are confined. For more general coefficients, the singular values would give rise to further zeros or poles of *y*_*n*+3_.

Next, we consider the situation in which one of *y*_*n*−1_ or *y*_*n*_ has either a zero or a pole at *z*_0_ and the other is finite and not equal to any of the other singular values: 0, *a*_±_*q*^2*n*^ or *b*_±_. Generically, these singularities belong to an infinite sequence of the form $\ldots ,0,k_{1},\infty ,k_{2},0,k_{3},\infty ,k_{4},\ldots$, where the *k*_*j*_s are finite and not equal to any of the other singular values. We now have enough information to calculate the degree of *y*_*n*_ for given generic initial conditions.

If *y*_0_ and *y*_1_ are generic rational functions, the singular values of one will not occur in the same locations as the singular points of the other. Furthermore, if *y*_0_ and *y*_1_ have degree one, then the zero and pole of *y*_0_ and the zero and pole of *y*_1_ determine four special points. Given an iterate *y*_*n*_, exactly one of its poles will occur at one of these special points and exactly one zero will occur at another. Let *N*_*n*_ be the number of new *b*_+_-points of *y*_*n*_, which is the same as the number of new *b*_−_-points as well as the number of new *a*_+_*q*^2*n*^-points and the number of new *a*_−_*q*^2*n*^-points. The poles of *y*_*n*+1_ come from the new *b*_+_- and *b*_−_-points of *y*_*n*_, apart from the single simple pole at one of the four special points. Hence, the degree *d*_*n*+1_ of *y*_*n*+1_ satisfies equation (2.2). Also, the *b*_+_-points of *y*_*n*_ are either new or they come from half the poles of *y*_*n*−1_ that are not at one of the special points. This gives us equation (2.3). Imposing the initial condition *d*_0_=*d*_1_=1 again gives us (2.4). For higher-degree generic initial conditions, the constant terms in equations (2.2) and (2.3) are replaced by bounded terms and the solution is seen still to grow like *n*^2^ for large *n*.

### (e) Other equations

In all examples that we have discussed so far, the entropy has been determined by considering the kind of singular behaviour that one considers in the traditional calculations used to determine singularity confinement. In these examples, there were also a finite number of points on the complex sphere where the initial conditions led to a different sequence of singularities but in the examples considered this contribution to the degree was small and so did not influence the entropy. This is not always the case.

Consider the equation

2.10 $$y_{n-1}+y_{n+1}=\sum_{k=0}^{K}a_{kn}y_{n}^{k},$$

for some integer *K*≥2, where *a*_*Kn*_≠0 for all *n*≥0. While there are simpler ways of calculating the degrees of iterates for this equation, we will continue with the same kind of analysis that we have applied to previous examples in order to illustrate the importance of looking at all singularities. First, notice that it is not possible for an iterate to become infinite at some point *z*_0_ if the previous two iterates were finite at *z*_0_. So if we choose to determine the degree by looking at the number of pre-images of ∞, we know that the location of the poles of any future iterate are the locations of the poles of the initial conditions *y*_0_(*z*) and *y*_1_(*z*). For example, suppose that *y*_1_ has simple a simple pole at *z*_0_ and *y*_0_ either has a simple pole or a regular point at *z*_0_. Then *y*_*n*_ has a pole of order *K*^*n*^ at *z*_0_. In particular, if *y*_0_(*z*) and *y*_1_(*z*) are degree one polynomials, then the degree of *y*_*n*_ (which we calculate using the only poles, which are at z=∞) is also *K*^*n*^ for *n*>0 and the entropy is log⁡K>0. This example again shows that we can still easily calculate degrees of iterates when singularities are not confined. However, unlike equation (1.1) for generic coefficients *a*_*n*_ and *b*_*n*_, the growth in degree is driven by a kind of periodic behaviour that does not usually play a role in traditional singularity confinement-type analysis.

## 3. Entropies for general initial conditions

In this paper, we have concentrated on determining the exact degree of *y*_*n*_ for given initial conditions, usually of degree one. The degree growth for more general initial conditions can easily be calculated and, moreover, bounds on the growth for arbitrary initial conditions can be obtained. It is possible of course to choose very special initial conditions such that the degrees grow slower than the generic case or even decrease rather than increase. In many cases however there will be a finite number of special singular points determined by the initial conditions, e.g. the point at infinity in equations (1.4) and (2.8), where certain singularities propagate but whose overall contribution amounts to a bounded term in the linear equation describing the degrees. The rest of the growth comes from calculating the number of new singular points as determined by the degree. The example (2.10) shows that sometimes the contribution of the special singular points can dominate the degree growth.

## 4. Conclusion

In this paper, we have shown through several examples that the standard singularity analysis that one performs in determining whether an equation possesses the singularity confinement property is almost sufficient, not only to calculate the entropy of the solutions but to calculate the exact degree of the *n*th iterate for given rational-in-*z* initial conditions. The results are both rigorous and elementary.

In a recent preprint, Ramani *et al.* [31] have built on ideas in this paper to develop an express method of integrability detection. They compare their method with their recently introduced de-autonomization approach. They apply their method to many interesting examples for which they are able to calculate the entropy exactly without the precise knowledge of the degrees.

The interpretation of the singularity analysis as a way of relating the multiplicities of various iterates at a point *z*_0_ is closely related to the complex-analytic analysis used in the estimates of the Nevanlinna characteristic. This idea played a central role in [32] where lower bounds on the growth of the Nevanlinna characteristic of meromorphic solutions were obtained using Nevanlinna's second main theorem and an assumption about the relative frequency with which certain singularities occur. These assumptions were dropped in future works [20,33,34] and the precise forms of the discrete Painlevé equations within the classes considered were obtained under the assumption that there is a meromorphic solution of finite order growing faster than the coefficients. In both the Nevanlinna approach and in the approach of this paper, slow growth is associated with a comparable number of singular values appearing in a sequence of iterates. Non-confinement typically means that we can find many more of one of the singular values than of another. However, as the example of Hietarinta & Viallet (1.5) shows, this can happen even when a singularity is confined. The calculation (2.7) shows that there are twice as many poles (counting multiplicities) than zeros, which ultimately leads to exponential growth.

Vojta's dictionary [35] related definitions and results in Nevanlinna theory to similar ideas in Diophantine approximation. The logarithmic height of a non-zero rational number *a*/*b*, where *a* and *b* are co-prime, is h(a/b)=log⁡max{|a|,|b|}. Applying this to the suggestion in [19] that difference Painlevé equations should have sufficiently many finite-order meromorphic solutions prompted the definition in [18] that a discrete equation is Diophantine integrable if the logarithmic height of the *n*th iterate is bounded by a power of *n*.

The initial papers [19,18] both only gave crude information about the form of low-growth (i.e. integrable) equations. This level of information was is in some sense comparable with the information one receives about the form of differential equations if one only considers the leading order behaviour of solutions in standard Painlevé analysis. More precise information comes from a detailed singularity analysis. In the context of height growth and Diophantine integrability, singularity calculations such as (1.2) can be reinterpreted as describing ‘closeness’ to certain values as measured by the different absolute values on Q, or more generally on a number field. The logarithmic height can be determined by knowledge of all absolute values. In this way, lower bounds on the height growth were determined in [22,23]. Connections between Nevanlinna theory, Diophantine integrability and the degree growth described in this paper are studied in [14] in analogues of the singularity confinement calculations are described in each setting for the same class of equations.
